# Supplementary figures and images for: The effect of the Wnt pathway on the osteogenic differentiation of periodontal ligament stem cells in different environments
Source: PeerJ. 2025 Jan 3;13:e18770. doi: 10.7717/peerj.18770 (PMC11702355; doi:10.7717/peerj.18770)

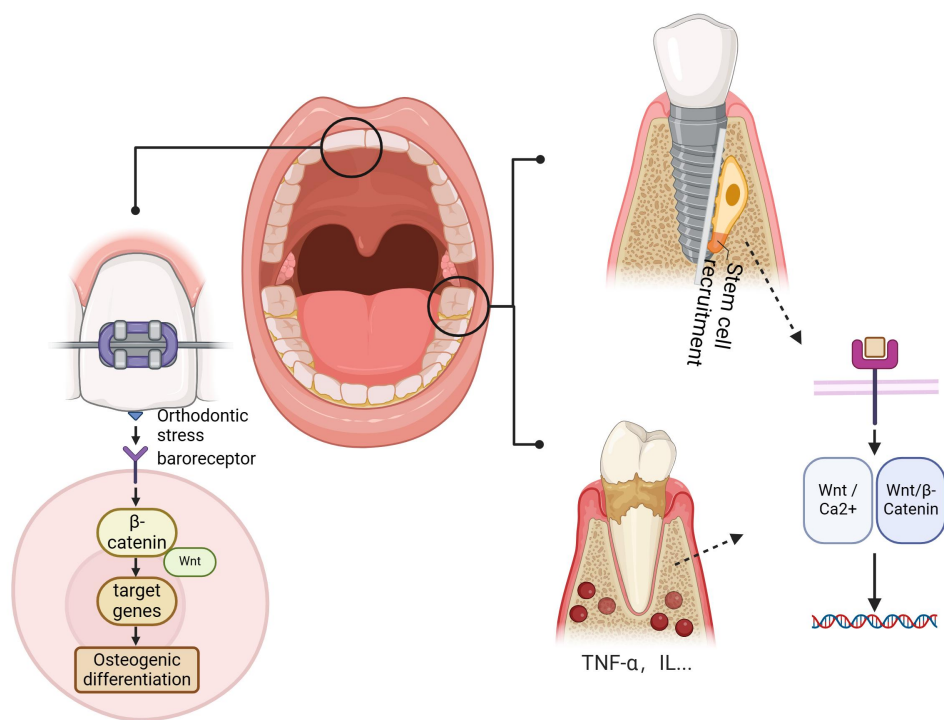

Supplement: Supplemental Information 1 — In different environments, PDLSCs located in the periodontal ligament affect Wnt signaling pathway through various factors, as well as osteogenic differentiation. Created in BioRender. [file peerj-13-18770-s001.pdf]
